# Catheter-related septic thrombophlebitis of the great central veins successfully treated with low-dose streptokinase thrombolysis and antimicrobials

**DOI:** 10.1186/1477-9560-3-11

**Published:** 2005-08-22

**Authors:** Patricia Volkow, Patricia Cornejo-Juárez, Ana Berta Arizpe-Bravo, Jorge García-Méndez, Enrique Baltazares-Lipp, Rogelio Pérez-Padilla

**Affiliations:** 1 Mexican National Institute of Cancer (INCan), Mexico City, Mexico; 2 Mexican National Institute of Respiratory Diseases (INER), Mexico City, Mexico

## Abstract

**Background:**

Septic thrombophlebitis is an iatrogenic life-threatening disease associated with use of central venous devices and intravenous (IV) therapy. In cancer patients receiving chemotherapy, vein resection or surgical thrombectomy in large central venous lines is time-consuming, can delay administration of chemotherapy, and therefore can compromise tumor control. Experience with thrombolysis has been published for catheter-related thrombosis but for septic thrombosis, this experience is scarce.

**Results:**

We describe three patients with cancer and septic thrombophlebitis of central veins caused by *Staphylococcus *aureus treated with catheter removal, thrombolysis, and intravenous (IV) antibiotics. In our reported cases, an initial bolus of 250,000 international units (IU) of streptokinase administered during the first h followed by an infusion of 20,000–40,000 IU/h for 24–36 h through a proximal peripheral vein was sufficient to dissolve the thrombus. After thrombolyisis and parenteral antibiotic for 4–6 weeks the septic thrombosis due to *Staphylococcus *aureus solved in all cases. No surgical procedure was needed, and potential placement of a catheter in the same vein was permitted.

**Conclusion:**

Thrombolysis with streptokinase solved symptoms, cured infection, prevented embolus, and in all cases achieved complete thrombus lysis, avoiding permanent central-vein occlusion.

## Background

Septic thrombophlebitis is an iatrogenic life-threatening disease associated with use of central venous devices and intravenous (IV) therapy. [1-3] Sole use of antimicrobials is rarely effective for controlling infection, requiring removal of the device and anticoagulation but in some cases a more aggressive approach such as resection of the affected vein [2,4-7] or trombectomy is needed [8]. Vein resection or surgical thrombectomy is time-consuming in large central venous lines, has a high rate of complications, can delay administration of chemotherapy, and therefore delay or impede tumor control. Experience with thrombolysis has been published for catheter-related thrombosis [9-13] but for septic thrombosis, this experience is scarce. [14,15] Herein, we describe three women with cancer and septic thrombophlebitis due to *Staphylococcus aureus* methicillin sensitive, who failed to resolve with catheter removal, parenteral antibiotics, and anticoagulation therapy and who were successfully treated with low-dose streptokinase fibrinolysis. All patients were receiving chemotherapy through non-tunneled polyurethane, single-lumen catheters placed in the subclavian-vein, but none of them had coagulopathy or septic shock.

## Results

### Case 1

**Figure 1:**
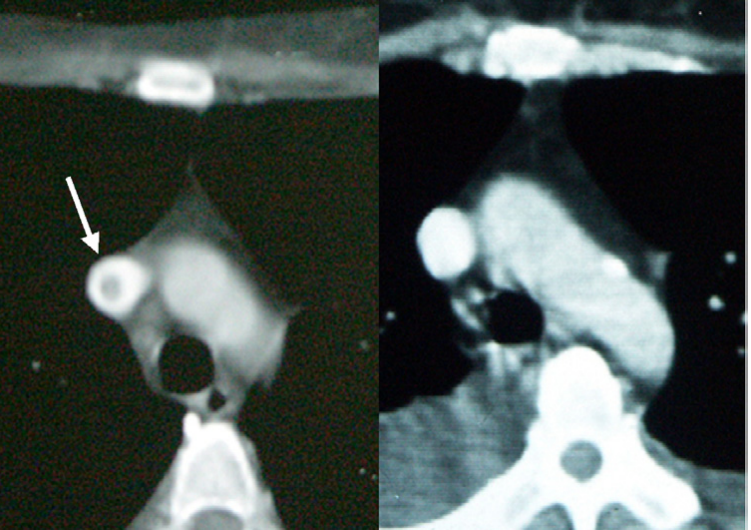
**CT scan showing thrombus before and after thrombolysis**. 1a. CT scan of superior vena cava with intravenous contrast infusion showing a thrombus before streptokinase infusion. 1b. CT scan of superior vena cava with intravenous contrast infusion three days after thrombolysis showing no remaining thrombus.

This was the case of a woman 59-years-of-age with papillary ovarian adenocarcinoma. She had been treated during the previous 10 years with 10 mg/day of prednisone for rheumatoid arthritis. A first central-vein catheter was placed for adjuvant chemotherapy and removed 4 months later with no complications. A new catheter was set in place 8 months later after documenting tumor relapse; however, one day later the patient developed pain at the insertion site and fever (39°C), and chills. An abscess at insertion site was found and the catheter was removed. Blood cultures, purulent secretion, and catheter tip were positive for *Staphylococcus aureus*. Intravenous dicloxacillin was initiated and amikacin was added 1 day later, but fever and positive blood cultures persisted. Echo-Doppler documented thrombosis of brachiocephalic trunk and computed tomography (CT) scan showed a thrombus reaching brachiocephalic trunk and superior vena cava (Figure 1a); subcutaneous (SC) enoxaparin was initiated. Vancomycin was started because fever and bacteremia persisted, with no clinical improvement. Seven days after beginning with antibiotics, the patient received an initial bolus of 250,000 international units (IU) of streptokinase administered in 1 h followed by an infusion of 40,000 IU per h for 24 h through a peripheral vein. One day after thrombolysis began, fever and positive blood cultures disappeared. Full permeability of right brachiocephalic vein and superior vena cava was documented by CT scan (Figure 1b). The patient completed 4 weeks of parenteral antibiotics, but died 1 month later with peritoneal carcinomatosis-related intestinal occlusion. Necropsy study showed neither thrombosis nor obstruction of great central veins.

### Case 2

A 42-year-old woman with breast cancer stage IIIB was started on chemotherapy. Four months later, she was admitted with fever, shivering, and painful erythematous lesions disseminated in legs and arms. The patient was initiated on IV vancomycin and amikacin, and after initial blood cultures grew oxacillin-sensitive *S. aureus*. The catheter was removed and antibiotics were changed to dicloxacillin and amikacin. Initial echo-Doppler for all limbs did not demonstrate obstruction to blood flow. Chest roentgenogram showed bilateral, multiple, rounded, irregular, non-cavitated opacities. Transthoracic echocardiography did not show heart valve vegetations, but did show a mobile hyper-reflectant image in superior vena cava extending to right atrium, suggestive of thrombus. Fever persisted and new painful nodular erythemathous lesions appeared in both limbs that evolved into abscesses, but neither purulent skin lesions nor blood cultures grew microorganisms. A second transthoracic echocardiogram performed 9 days later showed persistence of the same pediculated lesion that measured 40 × 10 × 10 mm. The patient was thrombolyzed with the same doses of streptotokinase; 24 h later, she had no fever, all symptoms resolved and transthoracic echocardiography performed 9 days later showed no lesions. She completed 6 weeks with IV antimicrobials. A month later, the scheduled mastectomy was performed the patient received 11 courses of weekly paclitaxel. Ten months later, the patient is asymptomatic with no evidence of tumor activity.

### Case 3

A 57-year-old woman with ovarian adenocarcinoma stage IV metastatic to lungs with a catheter placed in the right subclavian vein, through which she received four cycles of carboplatin and paclitaxel. One week after the last chemotherapy cycle, she developed fever and pain in the right shoulder and two days later presented to the emergency room. At admission, the patient had persistent shoulder pain, anorexia, and an enlarging, painful mass in right shoulder, with an indurated, extremely tender area in right sternoclavicular joint and edema in right arm. She was febrile, hypotensive, and tachycardic. Oxacillin-sensitive *Staphylococcus aureus *grew in blood and catheter tip, and was started on dicloxacillin and amikacin. Echo-Doppler revealed a 4-cm long thrombus within the right subclavian vein partially occluding the right jugular vein. No intracardiac thrombus or valvular lesions were observed in the Echocardiogram. CT scan showed a large collection of liquid in right shoulder sternoclavicular joint. The patient received an initial streptokinase bolus of 250,000 IU and one 12-h infusion of 20,000 U/h, followed by enoxaparin 60 mg BID. Twenty four hours later, Echo-Doppler showed patent right subclavian and jugular veins. She completed 3 weeks of enoxaparin and then changed to oral anticoagulation. Technetium bone scan showed evidence of ipsilateral clavicle osteomyelitis. She received IV antibiotics for 4 weeks followed by oral dicloxacillin plus rifampin for 28 weeks. After treatment the bone scan did not have evidence of osteomyelitis and 8 months later the patient had normal shoulder function without arm edema.

## Discussion

Intravascular infection and thrombosis are two of the most serious complications related to central venous catheter use. Central vein thrombosis was described as a complication of catheters in 1971 [2]. Symptoms were present in <4% of patients with central venous catheter when venography showed thrombosis [2]. Neoplastic disease often creates a thrombogenic state, through inflammation mediators, tumor necrosis factor, platelet activation, as well as a procoagulant substances released by tumor cells [15]. In addition, long indwelling lines increase risk for thrombosis, reported in 0.06-32% of patients, although the risk changes with type of catheter, neoplasia, chemotherapy regimes and radiation [16]. The complications of catheter-related thrombosis are similar although not as frequently as has been described for lower limb thrombosis [16]. It can produce pulmonary embolism. The trombus can become infected with persistent bacteremia and septic embolization ensue [17]. It has been recognized that CVC infection increases the risk of thrombosis [18] even though we believe that the incidence of septic thrombosis with persistent refractory bacteremia as the cases herein described is uncommon, in a recent review CVC associated thrombosis this complication is not mentioned [17]. For the last 6 years at a hospital where we placed >1,100 long indwelling catheters for cancer patients annually we have observed approximately one case every two years.

Standard therapy for catheter associated septic thrombosis includes antibiotics, catheter removal, full heparin anticoagulation, and venotomy. The latter is technically impossible for great central veins, although surgical thrombectomy has been successfully performed [8] and medical lysis of the thrombus is feasible [13,14]. We describe successful lysis of septic thrombosis with low-dose streptokinase infusion through a peripheral vein proximal to central great vein affected and no surgical or invasive procedure performed. This approach was first reported with a high percentage of success in catheter-related thrombosis in the early 1980s, allowing to maintain vein patency [4,6] using streptokinase, urokinase, and more recently, recombinant-tissue plasminogen activator, [13,14]. In our reported cases, streptokinase administered as initial bolus of 250,000 IU during 1 hour followed by infusion of 20,000-40,000 IU/h for 24-36 hours through a proximal peripheral vein was sufficient to dissolve the thrombus Table 1. This treatment solved symptoms, cured infection, prevented embolus, and in all cases achieved complete thrombus lysis, avoiding permanent central-vein occlusion. The episode of septic thrombosis due to *Staphylococcus aureus* solved with continued parenteral antibiotic for 4 to 6 weeks in all cases and no surgical procedure was required.

**Table 1:** Demographic and clinical characteristics of the patients described

Case	1	2	3
Gender	F	F	F
Age	59	42	57
Cancer site	Ovarian (relapse)	Breast stage III-B	Ovarian stage IV
Time of catheter stay (days) before symptoms	1	155	63
Thrombus site	Superior vena cava	Superior vena cava extended to right atrium	Subclavian and yugular veins
Days of antimicrobials before thrombolysis	7	19	6
Indication for thrombolyis	Persitent fever and bacteremia.	Persistent fever and septic embolization	Persitent fever and septic embolization
*Staphylococcus aureus *methicillin sensible	+	+	+
Streptokinase dose	250,000 IU hr. bolus + 40,000 us/hr for 24 hrs.	250,000 IU us/hr bolus.	250,000 us 1 hr. bolus + 25,000 us/hr for 12 hrs.
Thrombus lysis	100%	100%	100%
ARDS*	No	No	No

* Acute respiratory distress syndrome

## Conclusion

Fibrinolytic therapy with streptokinase is a therapeutic option in the management of catheter-related septic thrombophlebitis of the great central veins. This therapeutic approach, mantain central vein patency, allowing potential to place a new long indwelling catheter, the cornerstone for cancer patients who need chemotherapy.

## List of abbreviations

IV – intravenous

IU – International units

CT – Computed tomography

SC – subcutaneous

ARDS – Acute respiratory distress syndrome

## Competing interests

The authors declare that we have not competing interests in the interpretation of data or presentation of information influenced by our personal or financial relationship with other people or organizations.

## Authors' contributions

PV – Participated in the design of the study, collected data, wrote the manuscript, general supervision of the research group

PCJ – Collected data, wrote the manuscript

AAB – Collected data and looked for the researches made before related with the manuscript

JGM – Collected data and looked for the researches made before related with the manuscript

EBL – Revised the manuscript and analysis of data

RPP – Revised the manuscript, analysis of data and final approval of the version

All authors read and approved the final manuscript.
